# Glutathione imbalance in patients with X-linked adrenoleukodystrophy^☆^

**DOI:** 10.1016/j.ymgme.2013.05.009

**Published:** 2013-08

**Authors:** Sara Petrillo, Fiorella Piemonte, Anna Pastore, Giulia Tozzi, Chiara Aiello, Aurora Pujol, Marco Cappa, Enrico Bertini

**Affiliations:** a Unit for Neuromuscular and Neurodegenerative Diseases, Bambino Gesù Children's Hospital, IRCCS, Rome, Italy; b Laboratory of Metabolomics and Proteomics, Bambino Gesù Children's Hospital, IRCCS, Rome, Italy; c Catalan Institution of Research and Advanced Studies (ICREA), Barcelona, Spain; d Neurometabolic Diseases Laboratory, Bellvitge Biomedical Research Institute (IDIBELL), Hospitalet de Llobregat, Barcelona, Spain; e Centro de Investigación Biomédica en Red de Enfermedades Raras (CIBERER), ISCIII, Spain; f Institut de Neuropatologia, Bellvitge Biomedical Research Institute (IDIBELL), Hospital Universitari de Bellvitge, Universitat de Barcelona, Spain; g Unit of Endocrinology, Bambino Gesù Children's Hospital, IRCCS, Rome, Italy

**Keywords:** Glutathione, X-linked adrenoleukodystrophy (X-ALD), Oxidative stress, Redox markers

## Abstract

**Background:**

X-linked adrenoleukodystrophy (X-ALD) is a genetic disorder of X-linked inheritance caused by a mutation in the ABCD1 gene which determines an accumulation of long-chain fatty acids in plasma and tissues. Recent evidence shows that oxidative stress may be a hallmark in the pathogenesis of X-ALD and glutathione plays an important role in the defense against free radicals. In this study we have analyzed glutathione homeostasis in lymphocytes of 14 patients with X-ALD and evaluated the balance between oxidized and reduced forms of glutathione, in order to define the role of this crucial redox marker in this condition.

**Methods:**

Lymphocytes, plasma and erythrocytes were obtained from the whole blood of 14 subjects with X-ALD and in 30 healthy subjects. Total, reduced and protein-bound glutathione levels were measured in lymphocytes by HPLC analysis. Erythrocyte free glutathione and antioxidant enzyme activities, plasma thiols and carbonyl content were determined by spectrophotometric assays.

**Results:**

A significant decrease of total and reduced glutathione was found in lymphocytes of patients, associated to high levels of all oxidized glutathione forms. A decline of free glutathione was particularly significant in erythrocytes. The increased oxidative stress in X-ALD was additionally confirmed by the decrease of plasma thiols and the high level of carbonyls.

**Conclusion:**

Our results strongly support a role for oxidative stress in the pathophysiology of X-ALD and strengthen the importance of the balance among glutathione forms as a hallmark and a potential biomarker of the disease.

## Highlights

• All glutathione forms are abnormal in blood of X-ALD patients.
• Abnormal oxidative stress in X-ALD is confirmed by decrease of thiols in plasma.
• Abnormal oxidative stress in X-ALD is confirmed by increased carbonyls in plasma.
• Glutathione may be considered a redox hallmark/potential biomarker in X-ALD.

## 1 Introduction

It is well known that X-linked adrenoleukodystrophy (X-ALD) is the most common peroxisomal disorder [1]. The disease is caused by mutations in the ABCD1 gene that encodes for the peroxisomal membrane protein ALDP which is involved in the transmembrane transport of very long-chain fatty acids (VLCFA; = C22) [1–4]. A defect in ALDP results in elevated levels of VLCFA in plasma and tissues. Noteworthy, the accumulation of VLCFA in brain has been demonstrated to anticipate histopathological abnormalities and is a main inducing factor in the development of cerebral disease [5]. The clinical spectrum in males with X-ALD ranges from neurological asymptomatic patients, isolated adrenocortical insufficiency, and slowly progressive myelopathy to a devastating cerebral leukoencephalopathy [4–6]. The most frequentclinical forms are the childhood cerebral ALD (CCALD), adrenomyeloneuropathy (AMN) and isolated Addison disease, not associated to neurological symptoms. Cerebral ALD is the most rapidly progressive phenotype of X-ALD and manifests before 10 years of age leading patients to a vegetative state in few years. AMN manifests between 20 and 40 years of age by progressive paraparesis caused by long-tract axonopathy in the spinal cord [3]. Some AMN male patients will also develop cerebral demyelination with severe neuroinflammation.

The mouse model of X-ALD, a knockout of the ABCD1 protein, presents with a slow progressing, late onset axonal degeneration resembling human AMN [7,8]. In this model, oxidative stress and damage appear early, one year before disease onset [9]. The treatment with a combination of antioxidants containing α-tocopherol, N-acetyl-cysteine and α-lipoic acid reversed oxidative damage and energetic failure, together with the axonal degeneration and locomotor impairment in the mouse model of X-ALD [10,11]. Thus, oxidative stress seems to underlie axonal degeneration in adrenoleukodystrophy and represents a hallmark in the pathogenesis of X-ALD.

The purpose of this study is to analyze the dysregulation of the redox homeostasis in the blood of a series of X-ALD patients, with particular focus on the glutathione system, which provides the principal cellular protection against oxidative damage [12].

Total glutathione (Tot GSH) has several forms in cells, with the predominance of the reduced form (GSH). Glutathione exists also in two oxidized forms: the “free GSSG” and the “protein-bound GS-Pro”, whose modulation becomes critical under the conditions of oxidative stress. Besides the importance of the Tot GSH content in cell function, the cellular redox balance is essential so far, and it is assured by equilibrated ratios among the glutathione forms [12]. In this scenario, a decrease of GSH or an increase of the oxidized form GSSG reflects an oxidative perturbation of the cellular environment, whereas a variation of GS-Pro, which directly modulates important redox signaling pathways [13,14], may trigger a sequence of pathogenic events ultimately leading to metabolic diseases and/or their complications [15,16]. Indeed, all glutathione forms are differently modulated under the conditions of oxidative stress and all contribute to the health of the cell. GS-Pro, in particular, should be carefully considered, because it represents a mechanism of signal transduction by which cells respond effectively and reversibly to redox inputs and regulate most cellular pathways [13,14]. Generally, GS-Pro is undervalued because the processing techniques adopted in many laboratories lead to sample de-proteinization before glutathione analysis. This procedure deprives the sample of the protein-bound glutathione, which by far is essential in the faceted framework of the antioxidant response.

In this study, we have analyzed glutathione homeostasis in lymphocytes of 14 patients with X-ALD and evaluated the balance between oxidized and reduced forms of glutathione, in order to confirm that the dysregulation of this redox marker can be detected consistently in the blood of X-ALD patients and may be useful in monitoring clinical trials when using antioxidant therapies as a therapeutic strategy.

## 2 Materials and methods

### 2.1 Subjects

Different oxidative stress markers were determined in plasma, erythrocytes and lymphocytes of 14 patients with X-ALD (n = 4 with CCALD and n = 10 with AMN). The clinical characteristics of patients are summarized in Table 1. At diagnosis, the mean ± SD of VLCFA C26:0 was 3.23 ± 0.67 μM (normal range: 0–0.9 μM), whereas the mean ± SD of C26:0/C22:0 and C24:0/C22:0 ratios were 0.08 ± 0.03 μM (normal range: 0–0.02 μM) and 1.72 ± 0.24 μM (normal range: 0.74–1.3 μM), respectively.

### 2.2 Blood sample collection

Blood samples were collected into Vacutainer Tubes (Becton Dickinson, Rutherford, NY) containing EDTA and immediately processed. Whole blood was centrifuged at 450 ×*g*, for 3 min, to separate plasma from erythrocytes. Plasma was rapidly removed by aspiration and stored at − 80 °C until analysis. Erythrocytes were hemolyzed in distilled water and frozen at − 80 °C until determination of the antioxidant enzyme activities and GSH.

To prepare lymphocytes, whole blood was carefully layered on a gradient consisting of 3 ml Hystopaque-1119 and 3 ml of Hystopaque-1077 (Sigma-Aldrich, St. Louis, MI, USA) and centrifuged at 420 ×*g* for 40 min, at room temperature. Carefully removed by aspiration, the white band containing lymphocytes was collected into 15 ml conical plastic centrifuge tubes, diluted with 6 ml 0.9% NaCl, and centrifuged for 5 min at 420 ×*g*. The supernatant was discarded and the pellet suspended with 2 ml of ddH_2_O to lyse red blood cells. After hemolysis, 2 ml of 1.8% NaCl was added and sample centrifuged for 5 min at 420 ×*g* until obtaining a clear pellet of lymphocytes.

### 2.3 HPLC measurements of various glutathione forms

Immediately after preparation, washed lymphocytes were mixed with 100 μl of 10 mM phosphate buffer pH 7.2 (for free GSH), or with 100 μl of 10 mM phosphate buffer pH 7.2, containing 0.05 M N-ethylmaleimide (for GSSG and GS-Pro). Cells were then lysated by sonication three times for 2 s. After sonication, 50 μl of 12% sulfosalicylic acid was added, and the glutathione content in the acid-soluble fraction was determined. The protein pellet was dissolved in 150 μl of 0.1 M NaOH and the GS-Pro content was analyzed. Protein content was measured using the BCA-protein assay (Pierce, Rockford, Illinois, USA). All glutathione forms were measured as previously reported [17]. Briefly, 15 μl of 4 M NaBH_4_, 10 μl of 2 mM EDTA/DTT, 5 μl of 1-octanol and 10 μl of 1.8 M HCl were placed in the derivatization vial containing 15 μl of sample. After the mixture was incubated for 3 min, 50 μl of 1.5 M N-ethylmorpholine buffer pH 8.0, 200 μl of H_2_O_2_ and 10 μl of 25 mM bromobimane were added. After additional 3-min incubation, 20 μl of acetic acid was added and the mixture was injected into a 150 × 4.6 mm Hypersil-ODS column (Thermo Fisher Scientific, Bellefonte, PA, USA), equilibrated with 30 mM ammonium nitrate and 40 mM ammonium formate buffer, pH 3.6. S-bimane adducts were eluted from the column in 6 min with a gradient of acetonitrile, at a flow rate of 1.5 ml/min.

The HPLC system, with a sample processor and solvent delivery system, was an Agilent Technologies 1100 device equipped with a fluorescence detector G1321A operating at an excitation wavelength of 390 nm and an emission wavelength of 478 nm. Data were analyzed with the Agilent ChemStation software for Windows NT (Agilent Technologies, Waldbronn, Germany). Tot GSH amounts were calculated by adding free, oxidized and GS-Pro. GSH amounts were calculated subtracting GSSG from free glutathione concentrations.

### 2.4 GSH assay in plasma and red blood cells

GSH levels in plasma and red blood cells were determined by a spectrophotometric assay, as reported by Rahman et al. [18], with little modifications. Briefly, the assay is based on the reaction of GSH with 5,5′-dithiobis-(2-nitrobenzoic acid) (DTNB), which produces the oxidized glutathione-TNB adduct and TNB chromophore. The rate of formation of TNB is measured at 412 nm and is proportional to the concentration of GSH in the sample. The GS-TNB adduct is then reduced by Glutathione Reductase (GR) in the presence of NADPH, recycling GSH back into the reaction.

### 2.5 Plasma sulfhydryl content

The concentration of total thiols was measured in plasma by the colorimetric Ellman test using DTNB as chromogen [19] and expressed in μmol/ml.

### 2.6 Plasma carbonyl content

Plasma protein carbonyls were assayed according to the protocol provided by the manufacturer (Cayman Chemicals, Michigan, USA).

### 2.7 Antioxidant enzyme assays

SOD (EC 1.15.1.1) and GPx (EC 1.11.1.9) activities were spectrophotometrically assayed in the hemolyzed erythrocytes, as previously described [20]. SOD activity was expressed as the amount of protein causing a 50% inhibition of formazan dye (505 nm), employing xanthine and xanthine oxidase to generate superoxide radicals. Units of GPx activity were calculated following NADPH oxidation at 340 nm using cumene hydroperoxide as the substrate.

### 2.8 Very long chain fatty acids

VLCFA were measured according to the previously published method [21].

### 2.9 Statistical analysis

Statistically significant differences between groups were analyzed using Student's *t*-test for normally distributed variables and Mann–Whitney test for non-normally distributed variables. A value of p < 0.05 was considered statistically significant. A value of p < 0.001 was considered extremely statistically significant.

## 3 Results

A total of 14 patients with X-ALD were enrolled in this study. Mean age was 34.7 ± 17.8 years, (range between 6.2 and 64 years) for CCALD patients, the mean age was 14.7 ± 7.6 (range 6.2 to 24.3) and 42.7 ± 13.8 years (range 20.9 to 64) for AMN patients. The age, phenotype and ABCD1 gene mutations of patients are summarized in Table 1.

**Fig. 1:**
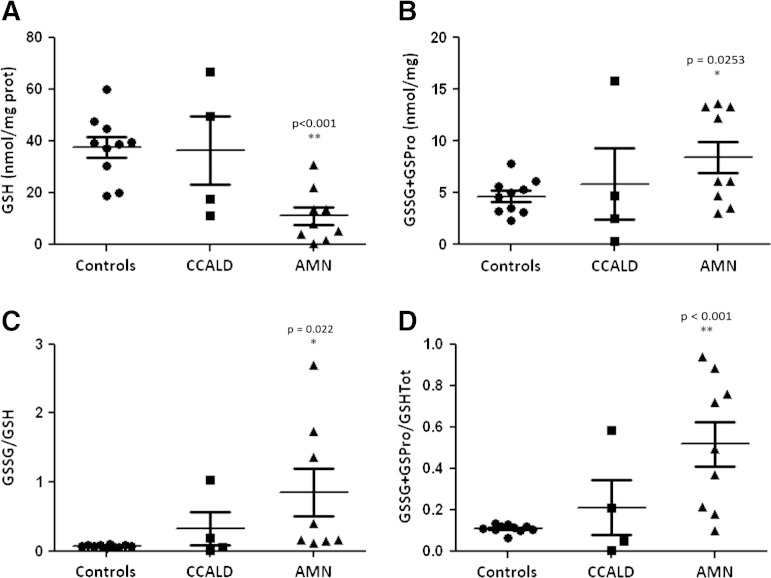
Lymphocyte glutathione levels in X-ALD patients. (A) Reduced glutathione is lower in patients than in controls (**p < 0.001). (B) All the oxidized glutathione forms (GSSG and GS-Pro) are significantly increased in patients (*p < 0.05). (C–D) Ratios between oxidized glutathione forms (GSSG, GS-Pro) and reduced or total glutathione are significantly increased in patients (*p < 0.05, **p < 0.001).

When compared to age-matched controls, lymphocyte concentration of the GSH was significantly decreased in AMN patients compared to controls (median value = 7.97 nmol/mg proteins, 95% confidence interval 0.86–27.40 vs median value = 39.10 nmol/mg proteins, 95% confidence interval 19.34–54.54, p < 0.001, Fig. 1A). No significant differences between CCALD and controls were observed. Conversely, all the oxidized glutathione forms (GSSG + GS-Pro) were significantly increased in AMN patients in relation to controls (median value = 6.13 nmol/mg proteins, 95% confidence interval 3.24–13.50 vs 4.8 nmol/mg proteins, 95% confidence interval 2.66–7.03, p < 0.05, Fig. 1B). Accordingly, the GSSG/GSH (median value = 0.28 nmol/mg proteins, 95% confidence interval 0.12–2.36 vs 0.07 nmol/mg proteins, 95% confidence interval 0.049–0.091, p < 0.05, Fig. 1C) and the GSSG + GS-Pro/Tot GSH ratios (median value = 0.49 nmol/mg proteins, 95% confidence interval 0.13–0.92 vs 0.11 nmol/mg proteins, 95% confidence interval 0.08–0.13, p < 0.001, Fig. 1D) were significantly increased, confirming the imbalance of the redox status in AMN patients.

**Fig. 2:**
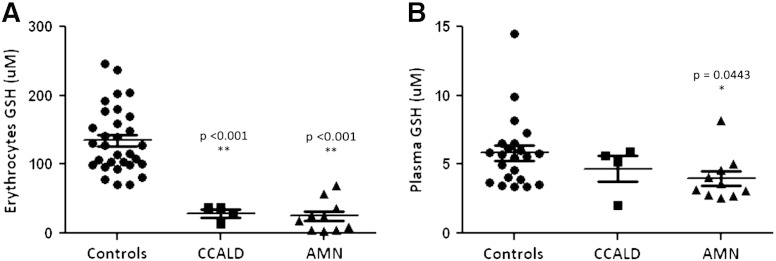
Erythrocyte (A) and plasma (B) GSH levels in X-ALD patients. *p < 0.05, **p < 0.001.

**Fig. 3:**
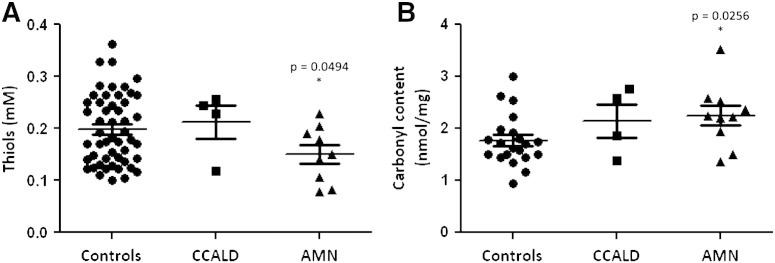
Plasma thiols (A) and carbonyls (B) in X-ALD patients. *p < 0.05.

As shown in Fig. 2, the GSH level in erythrocytes (median value = 20.91 μM, 95% confidence interval 4.42–63.42 vs 127.4 μM, 95% confidence interval 75.05–220.86, p < 0.001, Fig. 2A) and in plasma (median value = 3.37 μM, 95% confidence interval 2.62–6.76 vs 5.64 μM, 95% confidence interval 3.40–9.82, p < 0.05, Fig. 2B) of AMN patients confirmed the significant decrease obtained in lymphocytes. Interestingly, unlike lymphocytes and plasma, erythrocyte GSH is significantly reduced also in patients with CCALD (Fig. 2A). A further support to the presence of increased oxidative stress in X-ALD is also provided by plasma thiols, significantly reduced in patients (median value = 0.15 mM, 95% confidence interval 0.080–0.22 vs 0.18 mM, 95% confidence interval 0.11–0.31, p < 0.05, Fig. 3A) and plasma carbonyls, markers of irreversible protein damage, which were significantly higher in patients than in controls (median value = 2.23 nmol/mg proteins, 95% confidence interval 1.43–3.09 vs 1.70 nmol/mg proteins, 95% confidence interval 1.15–2.63, p < 0.05, Fig. 3B).

Concerning antioxidant enzymes, SOD and GPx activities were similar in patients and in controls (Table 2).

## 4 Discussion

Our results support previous experimental evidence that oxidative stress may play a significant role in the pathophysiology of X-ALD [9,10,22,23]. Oxidative modifications have been observed in postmortem X-ALD brains, where lipid peroxidation products mostly occur in the inflammatory demyelinative lesions and in adrenal cortex [24,25]. Oxidative damage has also been evidenced in skin-derived fibroblasts [9,26], plasma [27,28] and lymphoblasts [29], thus implying that oxidative stress may represent a systemic phenomenon linked to the loss of function of the ABCD1 transporter/ALD protein. Noteworthy, the plasma sulfhydryl content significantly increased in X-ALD patients after bone marrow transplantation (BMT) compared to controls, whereas no significant differences in plasma carbonyl content before and after BMT were found [28].

Oxidized proteins and lipid peroxidation-derived products have also been observed in a mouse model of X-ALD, where a combination of antioxidants (N-acetyl-cysteine, α-lipoic acid, and α-tocopherol) demonstrated to reduce oxidative markers and axonal degeneration in spinal cord [9–11]. However, despite promising results on the development of effective therapies with impact on the redox imbalance [30], so far neither the exact source of free radicals nor the molecular mechanism underlying oxidative damage has yet been elucidated in X-linked ALD. This information may be required to identify more sensitive and specific redox markers and to the development of tailored therapeutic strategies.

In this study, we show for the first time, that all glutathione forms are abnormal in the blood of X-ALD patients, including a profound decrease of GSH associated with high levels of the oxidized forms (GSSG + GS-Pro). Interestingly, the decline of free glutathione was particularly significant in erythrocytes. Erythrocytes are devoid of nucleus, which is critical in maintaining glutathione homeostasis in the cell. Therefore, the GSH differences between lymphocytes and erythrocytes may result from this important condition of red blood cells. Indeed, the nuclear glutathione has been demonstrated to affect its cytoplasmic levels and play an important role in oxidative signaling [31]. Under normal physiological conditions, the rate of GSH synthesis is finely regulated and multiple mechanisms are involved in its modulation both pre and post-transcriptionally. Key transcription factors, so far identified, include Nrf2/Nrf1, via the antioxidant response element (ARE), the activator protein-1 (AP-1) and the nuclear factor NFκB. Nrf2, in particular, is crucial in modulating GSH biosynthesis [32]. Upon recognition of signals imparted by oxidative and electrophilic molecules, Nrf2, located in cytoplasm, is released from its inhibitor (Keap1), escapes proteasomal degradation and translocates to the nucleus, where it induces the expression of the rate-limiting enzyme of glutathione synthesis (γ-glutamylcysteine synthetase, GCS) [33]. Thus, the contribution of the nucleus to the balanced levels of cellular glutathione is essential and erythrocytes, lacking of the nucleus, probably respond to oxidative insults with GSH fluctuations not promptly buffered by the glutathione synthesis, as instead occurs in nucleated cells as lymphocytes.

Noteworthy, low levels of GSH have recently been also reported in the spinal cord of Abcd1 null mice, well in advance of disease onset [10].

Glutathione is the major tissue redox buffer and it is a good indicator of the cellular redox environment, as it promptly responds to redox fluctuations [34]. GSH levels appear to play a vital role for X-ALD, as the patient's fibroblasts die when GSH biosynthesis is blocked with buthionine sulfoximine (BSO) [9]. Its depletion and ROS generation are highly interlinked, so it is difficult to determine which comes first in X-ALD pathogenic cascade. The decrease of GSH observed in our patients may be a consequence of the increased oxidative stress generated by VLCFA accumulation, which may be responsible of the early oxidative damage and represent the agent initiating oxidative stress. Indeed, membranes enriched with VLCFA are the earliest biochemical abnormality observed in the brain tissues of patients [35], and in X-ALD human fibroblasts, treated with an excess of both hexacosanoic acid and monounsaturated C26:1 fatty acid, ROS overload with GSH depletion has been observed [9,36]. Thus, VLCFA accumulation, due to mutations in ABCD1, may trigger the pathogenic cascade in X-ALD by oxidizing/inactivating the “energetic enzymes” of Krebs cycle and glycolysis, and leading to decreased levels of NADH and ATP, as shown in the spinal cords of X-ALD mice [10]. Glutathione homeostasis is strictly linked to NADH and ATP contents, as GSH needs ATP for its synthesis and a correct GSSG/GSH equilibrium depends on the action of the NADPH-consuming enzyme glutathione reductase. Therefore, the decrease of GSH levels may be a consequence of the VLCFA-induced oxidative stress, deriving from the imbalance among the NADH/NAD^+^ and/or NADPH/NADP^+^ redox-active cofactors [37], but may play itself an active role in X-ALD pathogenesis. Indeed, key enzymes of glycolysis and tricarboxylic acid cycle are sensitive to oxidation [38] and may represent additional targets for GSSG-driven oxidative damage in adrenoleukodystrophy.

Our data confirm and expand previous reports on the role of oxidative stress and damage in X-ALD, and pinpoint both a target cell type (erythrocytes) and a biomarker of potential use for monitoring clinical trials for X-ALD/AMN. Also, our results underscore the pertinence of using antioxidants aiming at replenishing GSH levels, such as N-acetyl-l-cystein, for therapeutic purposes in this disease, as proposed by Tolar et al. [39].

## 5 Conclusions

Accumulating evidence suggests a pivotal role for glutathione homeostasis in X-ALD and highlights the potential benefit of treatments addressing GSH imbalance, although additional studies and clinical trials are required. Maintaining balanced GSH levels may provide a potential therapeutic means in patients to slow down neurodegeneration and/or alleviating symptoms in all disease phenotypes.

## Figures and Tables

**Table 1:** Baseline characteristics of enrolled patients.

Subject	Age (years)	Phenotype	Mutation
HAAM	4	CCALD	c.1415_1416delAG (p.Q472RfsX83)
AM	24	CCALD	c.919C>T (p.Q307X)
SM	16	CCALD	c.1888G>A (p.E630K)
ON	11	CCALD	c.1628C>T (p.P543L)
MG	62	AMN	c.2006A>G (p.H669R)
AG	33	AMN	c.427C>T (p.P143S)
BM	64	AMN	c.1382delT (p.L461RfsX97)
PF	54	AMN	c.1252C>T (p.R418W)
RN	61	AMN	c.1415_1416delAG (p.Q472RfsX83)
ME	20	AMN	c.442_444 del 3(AAC)/ins6 (TGTTGA) (p.N148CfsX1)
SF	40	AMN	c.442_444 del 3(AAC)/ins6 (TGTTGA) (p.N148CfsX1)
LM	54	AMN	c.1540A>C (p.S514R)
LF	43	AMN	c.1415_1416delAG (p.Q472RfsX83)
LM	40	AMN	c.1415_1416delAG (p.Q472RfsX83)

**Table 2:** Summary of activities of antioxidant enzymes, SOD and GPx.

GPx (nmol/min/ml)	SOD (U/ml)	
AMN	864 ± 68	619 ± 62
CCALD	984 ± 116	526 ± 18
Controls	888 ± 74	571 ± 64
